# Microscopic Insight into Knudsen and Electromagnetic Effects on Thermal Conductivity of Closed Mesoporous Metal Gels

**DOI:** 10.3390/gels11090739

**Published:** 2025-09-15

**Authors:** Haiyan Yu, Ning Guo, Anqi Chen, Mingdong Li, Haochun Zhang, Mu Du

**Affiliations:** 1 Institute of Thermal Science and Technology, Shandong University, Jinan 250061, China; 202334300@mail.sdu.edu.cn (N.G.); 202234272@mail.sdu.edu.cn (A.C.); 202414341.sdu@vip.163.com (M.L.); 2 School of Energy Science and Engineering, Harbin Institute of Technology, Harbin 150001, China; hczhang@hit.edu.cn; 3 Institute for Advanced Technology, Shandong University, Jinan 250061, China

**Keywords:** thermal conductivity, microscale heat transfer, mesoporous metal gels, microscale thermal radiation

## Abstract

Accurate thermal characterization of closed mesoporous metal gels is vital for high-temperature uses, yet microscale effects often ignored in macroscopic models significantly impact heat transfer. This study introduces a new predictive method based on an equivalent Voronoi model, accounting for the Knudsen effect and microscale electromagnetic interactions. Predicted thermal conductivity closely matched experimental results, with an average error of 5.35%. The results demonstrate that thermal conductivity decreases with porosity, increases with temperature, and varies with pore size, with a minimum of 17.47 W/(m·K) observed at ~1 μm. Variations in refractive index, extinction coefficient, and specific surface area exert negligible influence. Conductive heat transfer is suppressed under Knudsen-dominated conditions at small pore sizes. Electromagnetic analysis around the pore size corresponding to minimum conductivity reveals localized surface plasmon resonances and magnetic coupling at the gas–solid interface, which enhance radiative dissipation and further reduce thermal conductivity. Radiation dissipation efficiency increases with decreasing porosity and pore size. This model thus serves as a predictive tool for designing high-performance thermal insulation systems for elevated-temperature applications.

## 1. Introduction

Thermal insulators with precisely engineered microstructures are pivotal to meeting current demands for energy efficiency and temperature regulation [1]. Among these, closed mesoporous metallic gels (CMM-gels) exhibit low effective thermal conductivity and robust thermomechanical stability [2,3], making them promising for applications such as aerospace thermal barriers, thermal management of power-dense electronics, renewable-energy conversion hardware, and high-temperature protective coatings [4,5]. To quantify heat transport in CMM-gels, researchers compute the effective thermal conductivity and decompose it into equivalent conductive and radiative components [6,7]. For closed pores smaller than approximately 4 mm, convective heat transfer is generally considered negligible [8].

Understanding thermal transport in the CMM-gels requires separate consideration of conductive (*κ*_cond_) and radiative (*κ*_rad_) pathways due to their distinct underlying physics. For *κ*_cond_, predictive approaches have evolved from empirically derived correlations [9], which are highly sensitive to specific material morphologies, toward physics-based methodologies. The latter include analytical thermal resistance network modeling via equivalent circuit methods [10], later refined to capture nanoscale solid-phase phonon scattering [10,11,12] and gaseous Knudsen effects [13,14] in mesoporous systems [11,15], as well as sophisticated numerical simulations employing Finite Element Analysis [16,17], Molecular Dynamics [18], or the lattice Boltzmann method [19,20] on reconstructed/idealized geometries. In contrast, *κ*_rad_ dominates at elevated temperatures and microscale/submicron characteristic dimensions [21,22], where surface roughness, intra-cavity scattering, and sub-wavelength near-field coupling [23,24] dramatically enhance non-contact heat transfer. This micro-radiative complexity fundamentally challenges conventional modeling paradigms: Full Radiative Transfer Equation (RTE) solvers [25,26,27] become computationally prohibitive beyond simplified geometries; approximations neglecting absorption/scattering [25] sacrifice physical fidelity; and the widely adopted Rosseland diffusion approximation [26,28] fails catastrophically as radiative fluxes exceed blackbody predictions at small scales. Attempts have been made to modify the Rosseland approximation by incorporating correction factors or accounting for near-field effects, but a unified approach remains elusive [14,25,29,30,31]. Consequently, developing a precise model for the radiative thermal conductivity of CMM-gels is essential.

This research provides a theoretically grounded and computationally efficient tool for predicting thermal transport in CMM-gels. The equivalent Voronoi model was used as a physical model, the conductive equivalent thermal conductivity was generated based on the molecular dynamics method, and the radiative thermal conductivity was derived from the microscale radiation method. Using this integrated prediction approach, seven critical factors influencing thermal conductivity—including porosity, cell size, temperature, the refractive index, the extinction coefficient, pore shape, and surface area to volume ratio—were systematically investigated. The study revealed fundamental relationships between these parameters and thermal transport characteristics, identifying optimal conditions for thermal conductivity minimization. The transmission mechanism of microscale thermal radiation was analyzed by examining electromagnetic distributions at critical configurations. Electromagnetic analysis demonstrates enhanced radiative dissipation efficiency through interfacial resonance phenomena at gas–solid boundaries, which actively suppress thermal conductivity. Then, the transmission mechanism of microscale thermal radiation inside the pores was analyzed by finding the electromagnetic distribution of the thermal conductivity minimum point in porosity and pore size.

## 2. Models

### 2.1. Geometric Model

For most mesoporous materials, the matrix comprises an assembly of numerous small unit elements [31]. Owing to their inherent structural irregularity and heterogeneity, developing an accurate theoretical model to predict the thermal conductivity of such materials remains highly challenging. To better replicate the internal architecture of CMM-gels, this study employs a complex three-dimensional Voronoi model to represent the closed-cell structure. Voronoi-based architectures have garnered significant attention for their capacity to capture the randomness and complex mesoscale morphology of real porous materials [32].

The foam geometry is defined by a Voronoi tessellation of randomly placed seed points, yielding polyhedral cells bounded by surfaces equidistant from neighboring seeds. Formally, the Voronoi region *V*(*pi*) associated with seed *p*_i_ is defined as [32]:

(1) $$V(pi)=\left\{p\right.\left|d\left(p,p_{i}\right)\right.\leq d\left(p,p_{j}\right),j\neq i,j=1,\ldots ,\left.n\right\}$$

where *V*(*pi*) denotes the Voronoi cell associated with seed point *pi* (with *i* being the index of this “center seed point”), *p* represents any spatial position vector, *d*(⋅,⋅) stands for the Euclidean distance metric, *n* corresponds to the total number of seed points in the domain, and *j* is the running index of all other seed points used for comparison in the Voronoi construction.

**Figure 1:**
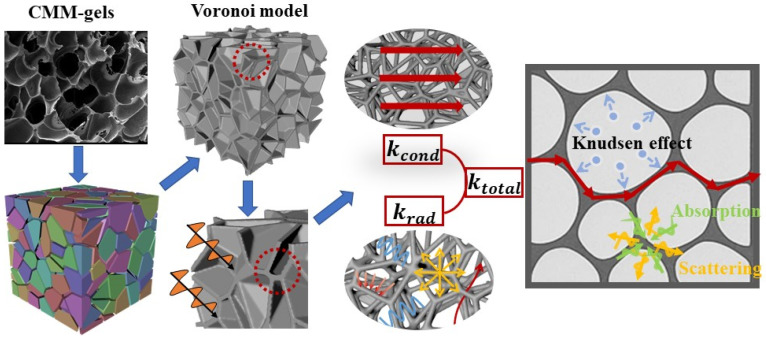
Schematic representation of the heat transfer model for CMM-gels.

Evaluating the pairwise distances among seeds thus produces an irregular polyhedral lattice that more accurately captures the geometric complexity of porous substrates. Subsequently, a cell wall thickness *d*_w_ is assigned to each Voronoi cell. During the construction process, the intersecting boundaries between adjacent cells are defined as solid walls, which serve as critical structural supports within the overall architecture, ultimately forming CMM-gels, as shown in Figure 1. In this Voronoi model, the porosity *φ* is defined as the ratio of the total air volume to the total volume of the geometric computational domain. The equivalent pore diameter *d*_p_ is determined based on the average pore size, calculated using the domain volume *V* and the total number of seed points *n*, following the relation *d*_p_ = *V*/*n*. The equivalent cell size, *D_h_*, is further defined as *D_h_* = *d*_p_/*φ*.

### 2.2. Calculation Methods

**Figure 2:**
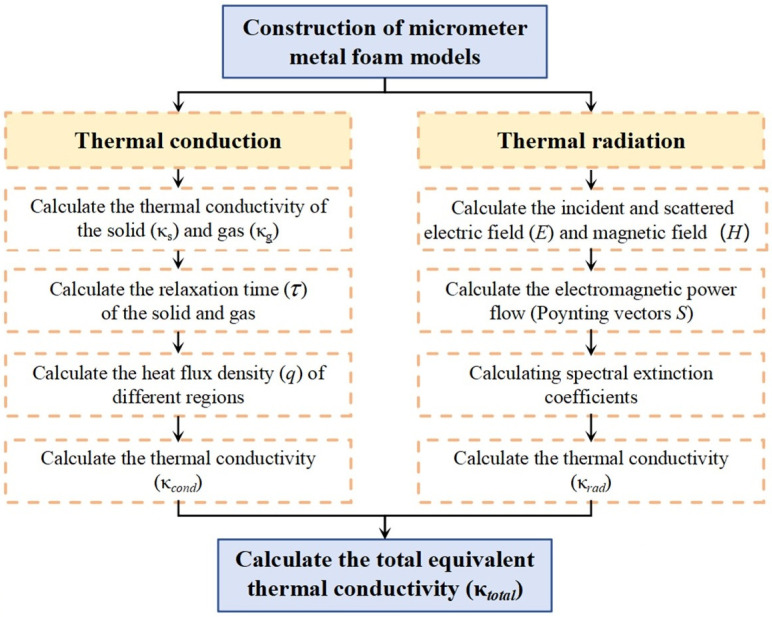
Flow diagram of the equivalent thermal conductivity calculation progress.

In this work, the CMM-gels were represented by a Voronoi model, and the effective thermal conductivity was determined by solving the governing heat conduction equations together with Fourier’s law. At micropore dimensions, conduction, radiation, and convection exhibit distinct behaviors, leading to significant variation in the overall thermal conductivity of porous materials [33,34]. When the characteristic pore diameter falls below 4 mm, density gradients in the confined air become negligible and the gas phase remains essentially stationary, so convective heat transfer can be neglected [35]. Consequently, only thermal conduction and thermal radiation are considered in the analysis. Therefore, the total effective thermal conductivity *κ*_total_ was expressed as follows [36]:

(2) $$\kappa_{total}=\kappa_{cond}+\kappa_{rad}$$

where *κ*_cond_ denotes the conductive contribution and *κ*_rad_ is the radiative contribution to effective thermal conductivity. The computational framework for predicting the effective thermal conductivity of the CMM-gels is illustrated in Figure 2.

In the present work, the conductive thermal conductivity $\kappa_{cond}$ is governed by Fourier’s law and the energy conservation equation, yielding a transient partial differential equation in space and time. The domain comprises a metal-skeleton region and a gas region, each with its own density, specific heat capacity, and thermal conductivity. Heat transport in both regions is described by the following conjugate heat transfer equations [37]:

(3) $${\left(\rho C_{P}\right)}_{s}\left(\frac{\partial T}{\partial t}\right)=k_{s}\nabla^{2}T\;\;\;\;\;,\;\;\;\;\;\;\;{\left(\rho C_{P}\right)}_{f}\left(\frac{\partial T}{\partial t}\right)=k_{f}\nabla^{2}T$$

where ρ denotes the density, $C_{P}$ represents the specific heat capacity, T is the local temperature, *t* stands for the time variable, $\nabla^{2}$ denotes the Laplace operator, while *k_s_* and *k_f_* correspond to the thermal conductivities of the solid skeleton and the gas phase, respectively. At the microscale, when the characteristic size of the cellular structure approaches the mean free path of gas molecules, the Knudsen effect becomes significant, leading to the suppression of gas-phase thermal conduction [22]. The Knudsen effect refers to the phenomenon in which, as the pore size decreases, the frequency of intermolecular collisions is reduced during heat transport within confined gas domains, resulting in a decline in thermal conductivity [38]. Therefore, in calculating the gas-phase thermal conductivity in the CMM-gels, the influence of both pore size and the mean free path of gas molecules must be considered. The corrected expression for gas-phase thermal conductivity that incorporates these microscale effects is given as follows [39]:

(4) $$k_{f}=\frac{k_{g0}}{1+2\beta K_{n}}$$

where *κ*_g,0_ is the bulk gas thermal conductivity (0.026 W·m^−1^·K^−1^ for air at 300 K and 1 bar) [40,41,42], and *β* is an energy accommodation coefficient (1.94 for air at 300 K and 1 bar) [39]. *Kn* = $\Lambda_{g}$/*d* is the Knudsen number, which refers to the ratio of the mean free path of the air $\Lambda_{g}$(68 nm for air at 300 K and 1 bar) [39] to the cell size *d*.

In this study, the lattice Boltzmann method (LBM) is employed as the numerical solver to simulate the thermal conduction process in CMM-gels, with the D3Q19 model being used for its high numerical stability and accuracy. In this approach the temperature field is represented by a set of distribution functions *f*_i_(*x*, *t*), each associated with one of nineteen discrete velocity vectors *e_i_*. Their evolution obeys the collision–streaming equation [39]:

(5) $$f_{i}\left(x+e_{i}\delta t,t+\delta t\right)-f_{i}\left(x,t\right)=-\frac{1}{\tau }\left[f_{i}\left(x,t\right)-{f_{i}}^{eq}\left(x,t\right)\right]$$

where *f*_i_ is the temperature distribution function in the *i*-th discrete direction at position *x*, δ*t* is the time step, *f_i_^eq^* represents the local equilibrium distribution function, *T*(*x*,*t*) is the local temperature, which is recovered as the zeroth moment, *T*(*x*,*t*) = ∑_i_ *f*_i_, and *τ* is the dimensionless relaxation time that differs for the gas and solid phases and can be expressed as follows [40]:

(6) $$\tau =\frac{3}{2}\frac{k_{s/f}}{\rho C_{P}c^{2}\delta t}+0.5$$

where *c* represents the phonon group velocity. Isothermal walls were imposed via the non-equilibrium bounce-back scheme [40], while adiabatic boundaries were handled by a reflective Neumann condition [41]. After the lattice Boltzmann solver reached steady state, the heat-flux density *q* was extracted [42] using the following:

(7) $$q=\frac{\tau -0.5}{\tau }\left(\sum e_{i}f_{i}\right)$$

The effective conductive thermal conductivity $\kappa_{cond}$ was then calculated as follows:

(8) $$\kappa_{cond}=\frac{L\times \int qdA\prime }{\Delta T\int dA\prime }$$

where *L* represents the length of the computational domain, and *A*′ is the cross-sectional area. In the following work, the $\kappa_{cond}$ of the CMM-gels was determined using Equation (9). The radiative thermal conductivity *κ*_rad_ of CMM-gels is obtained by treating thermal radiation as an electromagnetic phenomenon via Maxwell’s equations, which describe wave propagation and interaction with matter, as follows [42]:

(9) $$\nabla \times E=-\frac{\partial B}{\partial t},\;\nabla \times H=J+\frac{\partial D}{\partial t}$$

where *E* is the electric field, *H* the magnetic field, *B* is the magnetic flux density, *B* =*μH*, *μ* presents the permeability, *D* is the electric displacement vector, *D* = *εE*, *ε* is the permittivity, *J* is the current density, and *J* = *σE*, and *σ* is the electrical conductivity. Perfectly matched layers were applied at the domain boundaries to absorb outgoing waves and eliminate non-physical reflections. From the complex field solution using the finite element method (FEM), the time-averaged Poynting vector *S* can be obtained as follows [43]:

(10) $$S\;=\frac{1}{2}\cdot Re\{E\times H^{*}\}$$

The Poynting vector can be used to calculate the incident and scattered electromagnetic fields, from which the corresponding incident power can be obtained. The relationship of absorption coefficient *σ*_a,λ_ and the scattering coefficient *σ*_s,λ_ serves as the basis for further evaluating the thermal radiative properties of the material, as follows [44]:

(11) $$\sigma_{a,\lambda }=\frac{-Re\iint_{\Sigma }\left\{E^{\left(inc\right)}\times H^{\left(sca\right)*}+E^{\left(sca\right)}\times \left(H^{\left(inc\right)*}-H^{\left(sca\right)*}\right)\right\}\cdot ndA\prime }{N\cdot Re\{\left.E^{\left(inc\right)}\times H^{\left(inc\right)*}\right\}}$$

(12) $$\sigma_{s,\lambda }=\frac{Re\iint_{\Sigma }\left\{E^{\left(inc\right)}\times H^{(sca)*}\right\}\cdot ndA\prime }{N\cdot Re\{\left.E^{\left(inc\right)}\times H^{(inc)*}\right\}}$$

where *E^(sca)^* and *H^(sca)^* represent the scattered electric and magnetic fields, respectively, and *E^(inc)^* and *H^(inc)^* denote the incident electric and magnetic fields, respectively. *N* is the unit normal vector to the surface area, *R*e indicates the real part of a complex number, and * denotes the complex conjugate. {TC “2.3.2 Thermal radiation thermal conductivity calculation”\1 3} The extinction coefficient *σ*_e,λ_ is commonly used to characterize the overall energy loss during wave propagation and can be calculated as $\sigma_{e,\lambda }=\sigma_{s,\lambda }+\sigma_{a,\lambda }$. To further analyze the radiation mechanism of microscale thermal radiation, combined with the Beer–Lambert Law [45], the spectral absorptivity *A* can be obtained as $A=1-e^{-\sigma_{a,\lambda \cdot L}}$, and the spectral reflectivity *R*, calculated by $R=1-e^{-\sigma_{s,\lambda \cdot L}}$. In the Rosseland optical approximation, the Rosseland mean extinction coefficient *σ*_e,R_ is employed to describe the overall absorption and scattering characteristics of a material with respect to electromagnetic waves, particularly thermal radiation. It is obtained by performing a weighted average of the spectral extinction coefficients and the radiative intensity over all frequencies [46]:

(13) $$\frac{1}{\sigma_{e,R}}=\frac{\int_{0}^{\infty }\frac{1}{\sigma_{e,\lambda }}f\left(\lambda ,T\right)d\lambda }{\int_{0}^{\infty }f\left(\lambda ,T\right)d\lambda }$$

f(λ,T) represents the spectral distribution of Planck blackbody radiation and indicates the fraction of radiative energy at a given wavelength relative to the total emitted energy [46]. By incorporating the *σ*_e,R_ into the energy equation, the effective radiative thermal conductivity *κ*_rad_ can be calculated [47].

(14) $$\kappa_{\mathrm{rad}}=\frac{16\sigma_{\mathrm{SB}}{T_{m}}^{3}}{3\sigma_{e,R}}$$

where *σ*_SB_ denotes the Stefan–Boltzmann constant. The *κ*_rad_ in this paper is obtained by Equation (14); combined with Equation (8), the total thermal conductivity *κ*_total_ can be finally obtained. In order to analyze the proportion of thermal radiation and thermal conduction in more detail, the radiation contribution ratio is defined as ω = *κ*_rad_/*κ*_total_.

## 3. Results and Discussion

### 3.1. Model Verification

**Figure 3:**
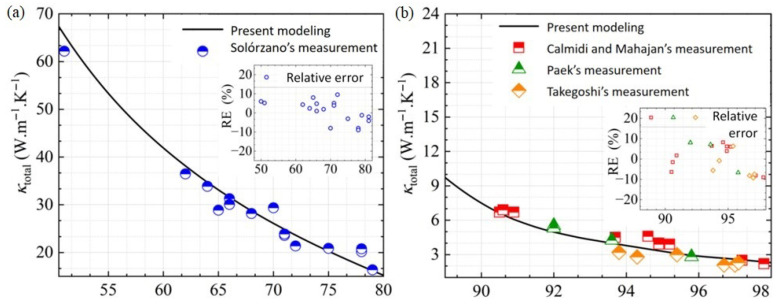
The comparison of the simulation results with the experimental data [48,49,50,51] under different processing: (**a**) powder metallurgy; (**b**) foaming.

To evaluate the reliability of the prediction model, the experimental data from the two most common processing methods currently used, powder metallurgy [48] and foaming [49,50,51], were compared and verified, encompassing porosity values between 50% and 98%. As illustrated in Figure 3, the simulation results exhibit a high degree of consistency with the experimental data [48,49,50,51], with an average error of 5.35% and maximum deviation below 8.6%. This deviation can be partially attributed to slight variations in porosity (±2%) and pore size distribution (±5%) inherent to the fabrication processes of porous metals, as reported in the original experimental studies [48,49,50,51], as well as minor simplifications in the Voronoi model’s geometric representation of complex real-world pore architectures. Moreover, the model accurately reproduces the observed dependence of thermal conductivity on porosity. These outcomes provide strong evidence for the credibility of the simulation method, which is subsequently adopted for detailed analysis of thermal transport in CMM-gels. Next, the impact factors’ effect on this predicted thermal conductivity is discussed in detail in the following section.

### 3.2. Factors Influencing the Equivalent Thermal Conductivity

This section systematically analyzes the influence of various factors on the heat transfer properties of CMM-gels. Specifically, the effects of cell size (*D_h_* = 0.1–100 μm), porosity (*φ* = 55–90%), temperature (*T* = 300–600 K), volumetric specific surface area (*VSSA*), refractive index *n*, and extinction coefficient *k* are investigated. Their impacts on the total thermal conductivity *κ*_total_, the radiative thermal conductivity *κ*_rad_, the conductive *κ*_cond_, and the radiative contribution ratio *ω* in CMM-gels are discussed in detail, specifically using aluminum properties as a base case, adopting the optical properties from Rakić’s experimental data [52].

#### 3.2.1. Effect of the Cellular Structure

**Figure 4:**
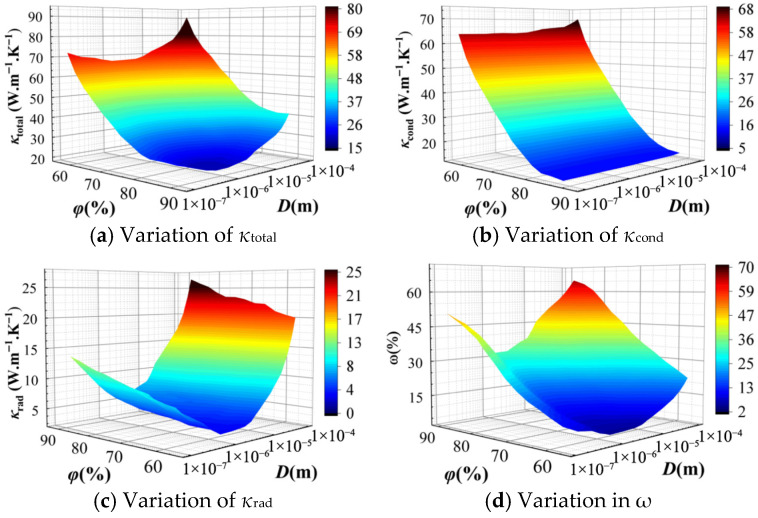
The thermal conductivity of CMM-gels versus cell size and porosity: (**a**) the total thermal conductivity; (**b**) the conductive thermal conductivity; (**c**) the radiative thermal conductivity; and (**d**) the radiative thermal conductivity proportion.

To investigate the effects of porosity and cell size on the thermal conductivity of CMM-gels, a total of 300 distinct models were generated with *T* = 300 K, 10 nm ≤ *D_h_* ≤ 100 μm, and 0.53 ≤ *φ* ≤ 0.94. The corresponding *κ*_total_, *κ*_rad_, *κ*_cond_, and *ω* are presented in Figure 4.

Figure 4a illustrates the variation in the *κ*_cond_ with respect to *D_h_* and *φ*. The overall trend indicates that *κ*_total_ decreases significantly as *φ* increases. This is attributed to the reduction in the solid framework, which enhances the relative influence of the gas phase. In addition, as the *D_h_* decreases, *κ*_total_ initially reaches a minimum, attaining its lowest value at *D_h_* = 1 μm, and then increases rapidly with further reduction in cell size. When the Knudsen effect is considered, Figure 4b presents that *κ*_cond_ decreases with increasing *φ* and decreasing *D_h_*. The Knudsen effect indicates that, at the microscale, the thermal conductivity of the gas phase is significantly suppressed due to the reduced frequency of intermolecular collisions, which limits the gas’s ability to transport heat. Consequently, smaller *D_h_* leads to greater dependence on the solid framework for heat conduction, resulting in a further reduction in *κ*_cond_. Moreover, in the closed cell, more intense collisions between gas molecules and the solid framework occur, leading to more pronounced variations in *κ*_cond_ compared to open-cell foams.

Figure 4c shows the variation in the *κ*_rad_ with the *D_h_* under high porosity conditions. It can be observed that *κ*_rad_ initially decreases with decreasing *D_h_*, reaches a minimum, and then increases as *D_h_* continues to grow. This non-monotonic trend is attributed to the influence of microscale effects on radiative transfer. As the *D_h_* decreases, particularly at the microscale, the radiative contribution becomes significantly enhanced. This enhancement arises because smaller *D_h_* shorten the radiative path length and intensify electromagnetic resonance within the material. This effect is especially pronounced when the structural dimensions approach the characteristic wavelength of Planck’s Law, where interference effects begin to dominate, leading to a marked variation in *κ*_rad_. The contribution of *κ*_cond_ to the *κ*_total_ exceeds that of the *κ*_rad_, as shown in Figure 4d. However, in high-porosity CMM-gels, as the cell walls become thinner and *φ* increases, *κ*_rad_ gradually becomes the dominant contributor to the overall thermal conductivity.

#### 3.2.2. Effect of Temperature

**Figure 5:**
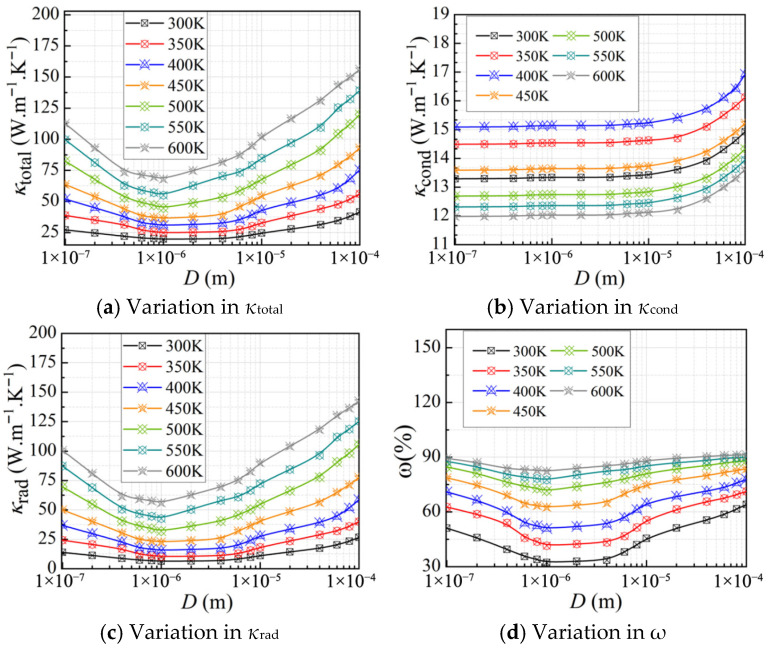
The equivalent thermal conductivity of CMM-gels versus cell size at different temperatures.

Since the CMM-gels are mostly applied with significant temperature changes, in this section, seven randomly generated models with *φ* = 90% were selected to investigate the influence of temperature on the *κ*_total_. The temperature range spans from 300 K to 600 K with an increment of 50 K. This analysis examines the variation of *κ*_total_ from room temperature to elevated temperatures. Based on experimental data [52], a temperature-dependent relationship for the thermal conductivity of solid aluminum was established, as illustrated in Figure 5.

As seen in Figure 5, when the *T* increases from 300 K to 600 K, both the *κ*_total_ and the *κ*_rad_ exhibit an increasing trend, which is consistent with the experimental results. *κ*_cond_ decreases with decreasing *D_h_*, after which the decreasing trend becomes less pronounced. This behavior is attributed to the Knudsen effect, where the reduced frequency of intermolecular collisions significantly suppresses the thermal conductivity of the gas phase. As the *D_h_* further decreases to the microscale or even nanoscale, the solid framework gradually dominates heat conduction, and the gas-phase contribution becomes negligible. Under these conditions, heat transfer is primarily governed by the solid matrix, leading to a slower variation in *κ*_cond_. With increasing *T*, the rise in *κ*_rad_ becomes more significant. This is due to the shift in the blackbody radiation peak toward shorter wavelengths (higher energy) at elevated temperatures, resulting in increased radiative energy and, consequently, a higher *κ*_total_. At a high *T*, *κ*_rad_ becomes the dominant contributor to the overall thermal conductivity. However, it is worth noting that when 0.6 μm < *D_h_* < 2 μm, the *κ*_total_ reaches a minimum and remains nearly constant over an extended interval. This observation suggests the existence of an optimal *D_h_* range, within which the *κ*_total_ is governed by a balanced interplay between *D_h_* and *φ*, resulting in a stable minimum value. In the production and application of the CMM-gels, precise control over *D_h_* is critical, which can help optimize *κ*_total_, enabling the development of materials with superior thermal insulation performance.

#### 3.2.3. Effect of Volumetric Specific Surface Area

**Figure 6:**
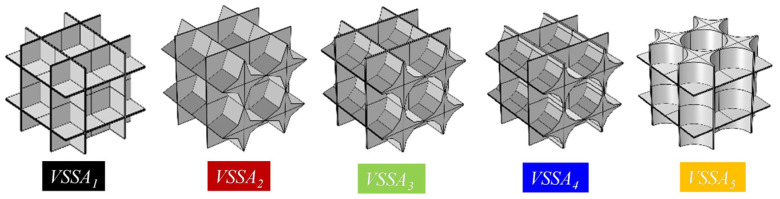
Illustration of volume-to-surface area ratios.

In these closed-pore models, the pore volume is maintained constant, and pores of different shapes are constructed within the CMM-gels to increase the overall surface area. As shown in Figure 6, when the *D_h_* = 100 μm, the models with different closed-pore shapes are a tetrahedron (surface area of 47,313 μm^2^), an octahedron (surface area of 45,538 μm^2^), a dodecahedron (surface area of 44,604 μm^2^), a tetradecahedron (surface area of 43,876 μm^2^), and a cylinder (surface area of 43,472 μm^2^). The volume-to-surface-area ratio (*VSSA*) of the models is obtained by comparing them with their volume. The order of volume-specific surface area from large to small is tetrahedron (*VSSA*_1_), octagonal prism (*VSSA*_2_), dodecaprism (*VSSA*_3_), tetradecephalone (*VSSA*_4_), and cylinder (*VSSA*_5_), to explore the effect of different volume specific surface areas on the equivalent thermal conductivity of CMM-gels. These models were calculated at *T* = 300 K and *φ* = 90%.

**Figure 7:**
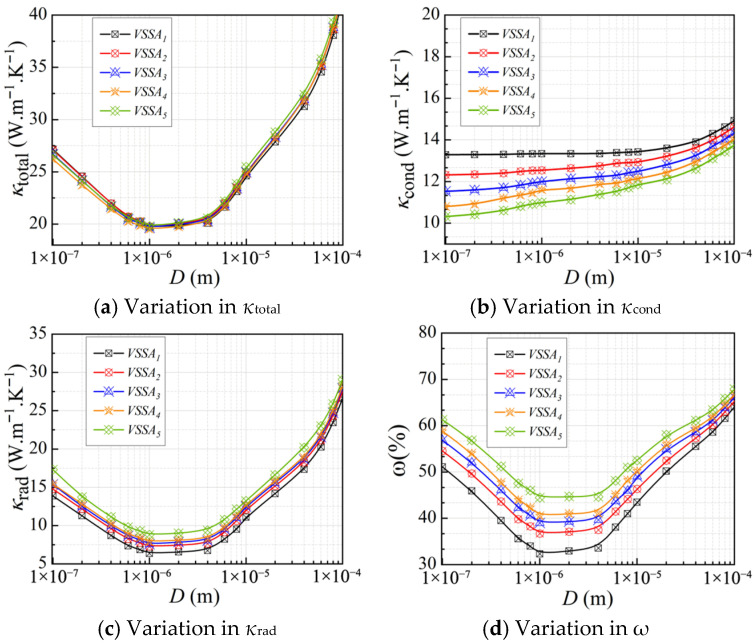
The equivalent thermal conductivity of CMM-gels versus cell size at different volume-to-surface area ratios.

By introducing serrated structures on the outer surfaces of pores in CMM-gels, as shown in Figure 7, the *VSSA* can be significantly increased, which in turn influences the *κ*_total_. At smaller *D_h_*, the increase in *VSSA* enhances the thermal conduction capability of the solid framework. This is because the serrated structures not only provide a larger solid surface area but also intensify scattering effects at the gas–solid interface. As a result, the efficiency of conductive heat transfer is increased, leading to an improvement in the *κ*_cond_, a phenomenon that becomes more pronounced as the characteristic size decreases. With increasing *VSSA*, the *κ*_rad_ exhibits a clear upward trend. The serrated structures enlarge the surface area of the solid matrix and introduce additional regions for absorption and scattering, thereby enhancing thermal radiation exchange at gas–solid interfaces. At the microscale, this structural optimization shortens the radiative transfer path and improves radiative efficiency, resulting in a significant increase in *κ*_rad_ with increasing *VSSA*. Under the combined influence of conduction and radiation, increasing the VSSA leads to an improvement in *κ*_total_, albeit only a slight one, which is likely due to the limited range of surface area values examined in this study.

#### 3.2.4. Effect of Material Properties

**Figure 8:**
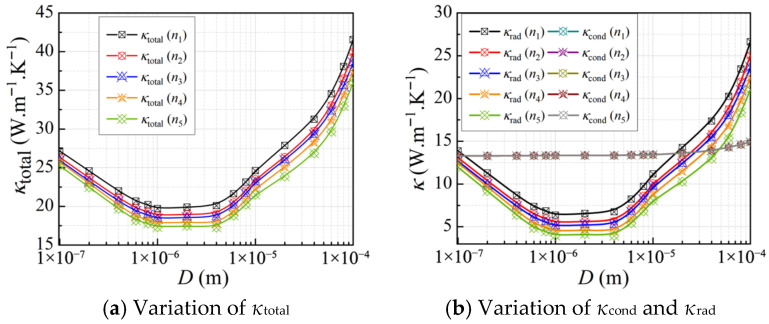
The equivalent thermal conductivity of CMM-gels versus the cell size at different refractive indexes.

For mesoporous materials, the radiative thermal conductivity *κ*_rad_ depends on the relative permittivity *ε* given by *ε* = (*n*+ i*k*)^2^, where *n* is the refractive index and *k* is the extinction coefficient [16]. In this study, *n* and *k* are varied independently to assess their separate effects on the *κ*_rad_, with one parameter held constant while the other changes with wavelength. In the following calculations, five models with different *n* and *k* were selected to examine the impact on the *κ*_total_ of CMM-gels at *T* = 300 K and *φ* = 90%. Here, *n*_1_ refers to the wavelength-dependent refractive index data from Rakić’s experimental data [52]. Similarly, *n*_2_, *n*_3_, *n*_4_, and *n*_5_ are defined as two, three, four, and five times the value of *n*_1_ at each wavelength, respectively, and the corresponding variation trends are shown in Figure 8.

Since changes in refractive index, as a component of relative permittivity, mainly influence electromagnetic wave propagation, their effect on conductive heat transfer is negligible. As shown in Figure 8, a higher *n* leads to greater internal refraction and reflection, making radiative transfer more sensitive to optical property variations. Thus, materials with higher *n* reflect more radiative energy, reducing transmission and the effective radiative path within the structure. This diminishes the efficiency of radiative heat transport and results in lower *κ*_rad_. When *n* is increased up to five times its baseline as *n*_5_, *κ*_total_ consistently reaches a pronounced minimum within the *D*_h_ range of approximately 0.6 μm to 2 μm, forming a stable conductivity valley, remaining large changes in *n*.

**Figure 9:**
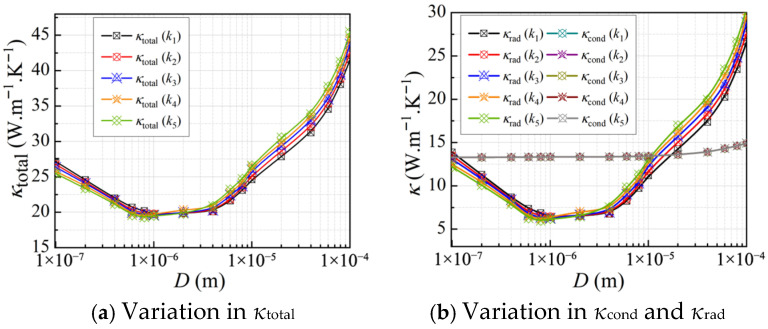
The equivalent thermal conductivity of CMM-gels versus the cell size at different extinction coefficients.

Likewise, five model groups with extinction coefficients are set separately; *k*_1_ denotes the extinction coefficient data from Rakić’s experimental data [52] as a function of wavelength, while *k*_2_, *k*_3_, *k*_4_, and *k*_5_ represent two, three, four, and five times *k*_1_ at each wavelength, and the corresponding variation trends are shown in Figure 9.

At the microscale, the effect of the *k* on the *κ*_rad_ shows a distinct dependence on the *D*_h_. When the *D*_h_ > 10 μm, increasing the *k* leads to higher *κ*_rad_. Conversely, as the *D*_h_ decreases, a higher *k* results in a reduction of *κ*_rad_. This phenomenon is attributed to the fact that larger *D*_h_ values provide longer radiative transfer paths, allowing radiation to propagate more efficiently and thereby enhancing *κ*_rad_. In contrast, when *D*_h_ is small, the shortened path increases the frequency of internal reflection and scattering, which confines thermal energy within the reduced *κ*_rad_.

### 3.3. Spectral Radiative Properties

**Figure 10:**
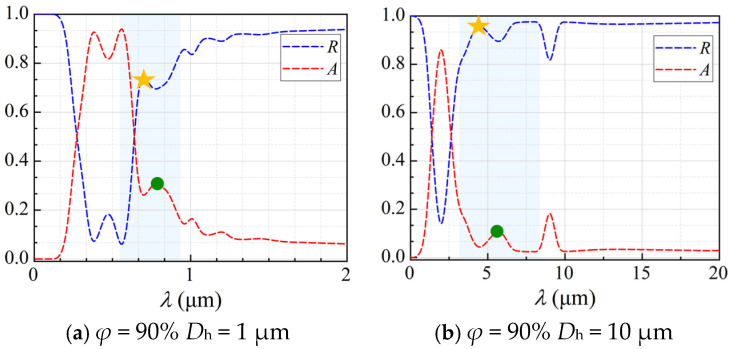

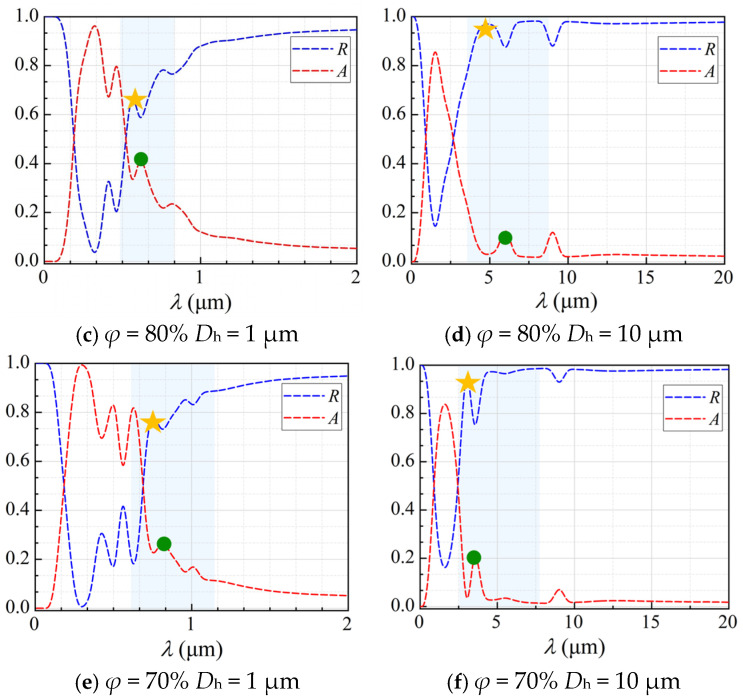
The spectral radiative properties of CMM-gels at minimum *κ*_total_.

To investigate the thermal radiative properties of the CMM-gels, a series of regular models were constructed based on the occurrence of minimum *κ*_total_ at *D*_h_ = 10 μm and *D*_h_ = 1 μm, under porosities of *φ* = 90%, *φ* = 80%, and *φ* = 70%, respectively. The spectral radiative properties, including absorptivity *A* and reflectivity *R*, were calculated over the visible and infrared wavelength ranges, with the results as shown in Figure 10.

As shown in Figure 10, the CMM-gel structures exhibit consistent spectral trends across all wavelengths. For example, at porosities of 90% and 80% with *D*_h_ = 1 μm, both *R* and *A* display pronounced oscillations below 2.5 μm. In the CMM-gels, *A* is high in the short-wavelength region and then decreases with fluctuations as the wavelength increases, while *R* transitions from an initial minimum to near-total reflection at longer wavelengths. For *D*_h_ = 10 μm, similar behavior is observed, with *A* rising sharply before 2.5 μm and then dropping, and *R* showing a corresponding increase. At longer wavelengths, both *A* and *T* are negligible compared to *R*. These results demonstrate the strong spectral tunability of CMM-gel structures.

**Figure 11:**
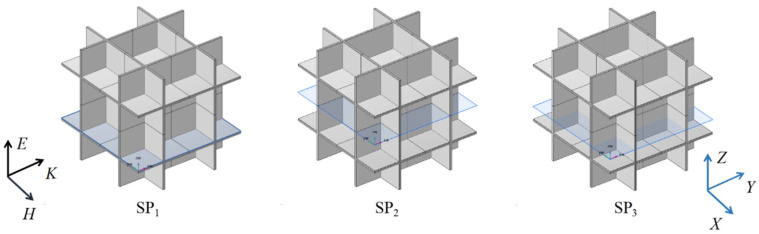
Cross-section schematic.

To further clarify the mechanisms underlying the observed spectral peaks, models with *D*_h_ values of 10 μm and 1 μm and porosities of 90%, 80%, and 70% were analyzed, as shown in Figure 11. For *D*_h_ = 1 μm, electric and magnetic field distributions were extracted at *Z* = 0 μm, 0.5 μm, and 1 μm; for *D*_h_ = 10 μm, field distributions were obtained at *Z* = 0 μm, 5 μm, and 10 μm.

**Figure 12:**
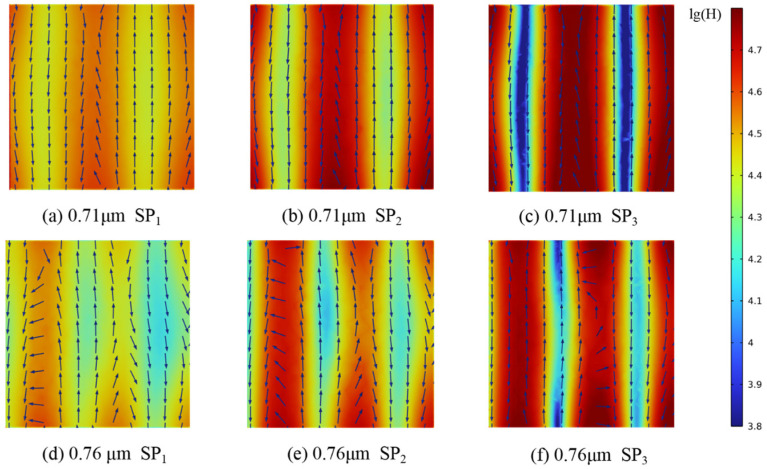
Magnetic field strength and electric field distribution; *φ* = 90%, *D_h_* = 1 μm.

**Figure 13:**
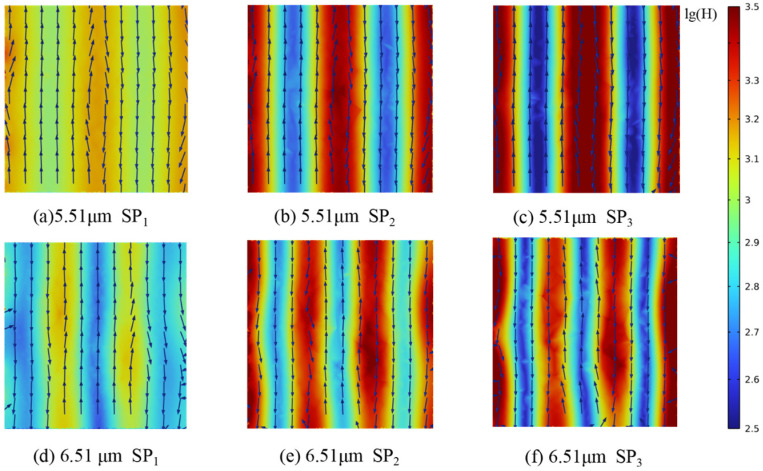
Magnetic field strength and electric field distribution; *φ* = 90%, *D_h_* = 10 μm.

**Figure 14:**
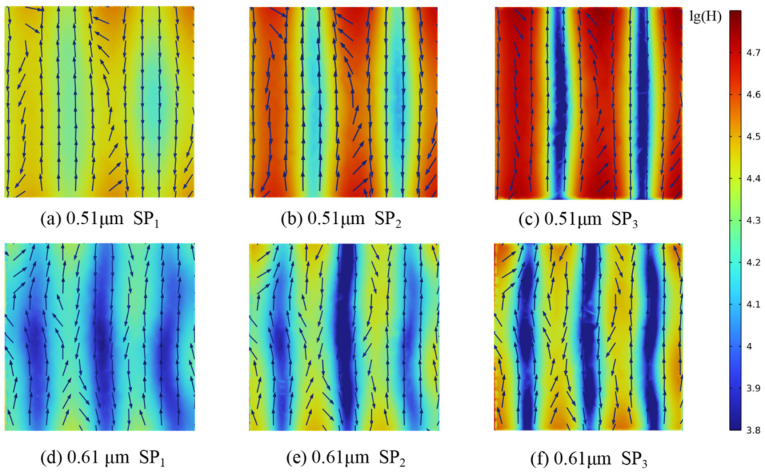
Magnetic field strength and electric field distribution; *φ* = 80%, *D_h_* = 1 μm.

**Figure 15:**
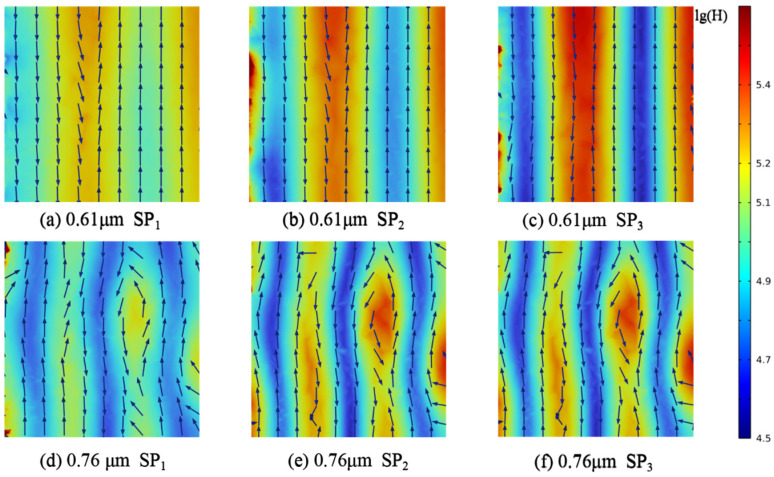
Magnetic field strength and electric field distribution; *φ* = 70%, *D_h_* = 1 μm.

**Figure 16:**
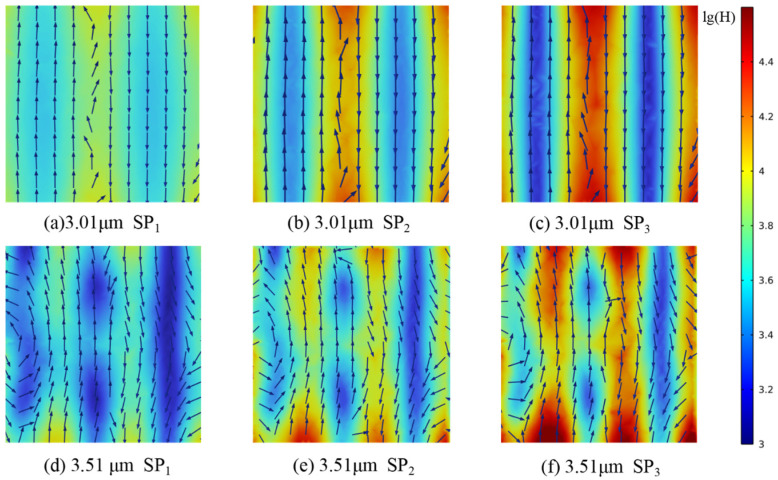
Magnetic field strength and electric field distribution; *φ* = 70%, *D_h_* = 10 μm.

The positions of the *R* and *A* peaks within the computational domain were determined by tracking the wavelengths at which these maxima occur. Changes in magnetic field intensity and electric field orientation were recorded from the pore edge to the center. Furthermore, analysis of the energy flux at the gas–solid interface enabled the calculation of radiative dissipation efficiency, as illustrated in Figure 12, Figure 13, Figure 14, Figure 15 and Figure 16.

Magnetic field enhancement regions are always associated with changes in electric field direction, which appear throughout the interior of the closed-cell structure due to the combined effects of surface plasmon resonance and magnetic resonance. Surface plasmon resonance at the gas–solid interface concentrates electromagnetic energy and deflects the electric field, while magnetic resonance causes local suppression of the magnetic field. These phenomena produce characteristic field patterns and magnetic field bands, as illustrated in Figure 12, Figure 13, Figure 14, Figure 15 and Figure 16. In CMM-gels, most variations in electromagnetic field intensity and direction occur inside the structure. For quantitative comparison, the radiative dissipation efficiency *η*_rad_ was denoted as *η*_rad_ = *S*_s_/*S*_m_, which is calculated as the ratio of the internal gas-phase energy flux *S*_s_ to the total cross-sectional energy flux *S*_m_, enabling direct quantitative comparison across different model configurations. The *η*_rad_ of each cross-section is shown in Table 1.

As shown in Table 1, analysis of structures with varying *D*_h_ and *φ* shows that as *D*_h_ decreases, electromagnetic wave propagation paths shorten, leading to more concentrated magnetic field enhancement and more frequent electric field perturbations. The number of pronounced magnetic field bands also increases in smaller *D*_h_. Moving from the edge to the center of the cross-section, the variation in the electromagnetic field becomes more intense, indicating that radiative effects are strongest near the central region of the structure. By comparing the *η*_rad_ among the models, it is observed that higher *φ* results in a higher *η*_rad_, while at the same *φ*, larger *D*_h_ yields higher *η*_rad_. Enhanced internal radiative dissipation reduces *κ*_total_ by increasing thermal energy loss. These findings confirm that microscale phenomena such as surface plasmon resonance and magnetic resonance are critical for tuning heat transfer properties in CMM-gels and that structural design and material selection enable precise control of both thermal conductivity and electromagnetic response for advanced thermal management applications.

## 4. Conclusions

In this study, a numerical method combining a Voronoi-based prediction model, the Knudsen effect, and microscale electromagnetic interactions was developed to investigate the effective thermal conductivity and internal heat transfer mechanisms of closed mesoporous metal gels. The model predictions showed close agreement with experimental data, with a maximum deviation of 8.6% and an average error of 5.35%. Systematic parametric analysis revealed clear dependencies of effective thermal conductivity on porosity, pore size, temperature, and optical parameters. Electromagnetic field simulations further identified that surface plasmon resonance and magnetic resonance at the gas–solid interface play a key role in enhancing radiative dissipation. The main findings are as follows:

- The developed numerical model predicts the effective thermal conductivity, within 8.6% error compared with experimental measurements.
- Effective thermal conductivity decreases with increasing porosity, increases with temperature, and reaches a stable minimum at a pore size of about 1 μm. The effects of refractive index, extinction coefficient, and surface area are comparatively minor.
- Electromagnetic resonance phenomena, including surface plasmon and magnetic resonance at the gas–solid interface, significantly enhance internal radiative dissipation and further reduce thermal conductivity.

These effects are amplified in structures with higher porosity and smaller pores, offering clear guidance for the design of next-generation thermal insulation materials.

## Figures and Tables

**Table 1:** Radiation dissipation efficiency *η*_rad_ at different cross-sections.

*λ*	SP_1_	SP_2_	SP_3_	*φ*	*D*_h_
0.71 μm	24.43	29.13	36.75	90%	1 μm
0.76 μm	30.88	34.43	42.78		
5.51 μm	10.72	12.46	23.28		10 μm
6.51 μm	20.69	22.34	29.90		
0.51 μm	27.24	32.53	42.16	80%	1 μm
0.61 μm	28.87	36.39	49.07		
5.01 μm	18.07	23.3	30.33		10 μm
6.01 μm	22.73	27.84	33.39		
0.61 μm	29.42	34.71	45.66	70%	1 μm
0.76 μm	31.34	36.75	49.43		
3.01 μm	18.27	26.13	38.37		10 μm
3.51 μm	24.87	29.67	45.87		

## Data Availability

The original contributions presented in this study are included in the article. Further inquiries can be directed to the corresponding authors.
